# RIOK3-Mediated Akt phosphorylation facilitates synergistic replication of Marek’s disease and reticuloendotheliosis viruses

**DOI:** 10.1080/21505594.2022.2096247

**Published:** 2022-07-26

**Authors:** Xusheng Du, Defang Zhou, Jing Zhou, Jingwen Xue, Ziqiang Cheng

**Affiliations:** College of Veterinary Medicine, Shandong Agricultural University, China

**Keywords:** MDV, REV, synergistic replication, RIOK3, Akt

## Abstract

Co-infection of Marek’s disease virus (MDV) and reticuloendotheliosis virus (REV) synergistically drives disease progression, yet little is known about the mechanism of the synergism. Here, we found that co-infection of REV and MDV increased their replication via the RIOK3-Akt pathway. Initially, we noticed that the viral titres of MDV and REV significantly increased in REV and MDV co-infected cells compared with single-infected cells. Furthermore, tandem mass tag peptide labelling coupled with LC/MS analysis showed that Akt was upregulated in REV and MDV co-infected cells. Overexpression of Akt promoted synergistic replication of MDV and REV. Conversely, inhibition of Akt suppressed synergistic replication of MDV and REV. However, PI3K inhibition did not affect synergistic replication of MDV and REV, suggesting that the PI3K/Akt pathway is not involved in the synergism of MDV and REV. In addition, we revealed that RIOK3 was recruited to regulate Akt in REV and MDV co-infected cells. Moreover, wild-type RIOK3, but not kinase-dead RIOK3, mediated Akt phosphorylation and promoted synergistic replication of MDV and REV. Our results illustrate that MDV and REV activated a novel RIOK3-Akt signalling pathway to facilitate their synergistic replication.

## Introduction

Both Marek’s disease virus (MDV) and reticuloendotheliosis virus (REV) are important oncogenic viruses that cause immunosuppression and tumours in chicken flocks, leading to significant economic losses in the poultry industry [1,2]. In addition to single-infection, numerous studies have reported the simultaneous infection of MDV and REV in chicken flocks [3–15]. Co-infection of MDV and REV alters the biological characteristics, pathogenicity, and epidemiologic status of the two viruses and modulates the immune response and host susceptibility. The integration of the partial or full REV genome into MDV is a common phenomenon in MDV and REV co-infected cells [16–18]. These recombination events can alter the biological functions of MDV and REV [19–25], which could promote the transmission and pathogenicity of the two viruses [26–29]. Furthermore, MDV and REV co-infection significantly enhance disease severity and decrease the antibody levels elicited by MD vaccines, consequently increasing susceptibility to secondary infections [30,31]. In addition, REV can be transmitted by inoculation with contaminated MD vaccines [32–35]. Despite MDV and REV co-infection events in the poultry being increasingly detected, little is known about the synergistic mechanism of the two viruses.

Viruses generally alters a variety of cellular functions and pathways to replication. The phosphatidylinositol-3-kinase-Akt (PI3K/Akt) pathway has been shown to play crucial roles in virus replication in both single- or co-infection cases [36–38]. Phosphorylated Akt was identified as a centre regulator in PI3K/Akt pathway to trigger virus replication [39,40]. In most cases, phosphorylation of Akt was associated with activated PI3K. The active PI3K is recruited to the membrane and catalyzes phosphatidylinositol-3,4-bisphosphate (PIP2) to generate phospholipid phosphatidylinositol-3,4,5-triphosphate (PIP3) [39]. Thereafter, serine/threonine-protein kinase Akt and phosphoinositide-dependent protein kinase (PDK1) binds to PIP3 on the plasma membrane. Firstly, PDK1 phosphorylates serine/threonine-protein kinase Akt at threonine 308 and then mTORC2 phosphorylates Akt at threonine 473. Activated Akt can phosphorylate eukaryotic initiation factor 4E-binding protein 1 (4EBP1) through activate mTORC1 to stimulate cellular translation. It can also activate anti-apoptotic transcription factor FoxO1 and several targets which mediate proliferation [39–41].

It has been demonstrated that Akt activity modulated by many viruses for replication function. HIV inhibit premature apoptosis by inducing Akt activity to facilitate virus replication, and herpes simplex virus 1 (HSV-1) replication also benefit from Akt phosphorylation [42,43]. In viral co-infection, HIV Nef synergizes with Kaposi’s sarcoma-associated herpesvirus (KSHV) vIL-6, which results in the activation of the Akt pathway, enhancing angiogenesis and tumorigenesis [38]. For some viruses, the activation of Akt, but not PI3k, plays an important role in viral replication. Akt phosphorylates the phosphoprotein of non-segmented negative-strand RNA viruses, driving RNA-dependent RNA polymerase activity [44,45].

RIOK3 (right open reading frame kinases 3) is a conserved atypical serine/threonine protein kinase within the RIO kinase family. It was reported that RIOK3 is an oncogene in breast cancer, glioma, pancreatic cancer and prostatic cancer through a variety of regulatory mechanisms [46,47]. RIOK3 was also found to regulate the type I IFN pathway during viral infection [48] and play as a component of pre-40S pre-ribosomal particles [49].

A recent study demonstrated that MDV activates the PI3K/Akt pathway, leading to reduced host cell apoptosis and increased virus replication [50]. However, there is no information on the role of Akt activation in MDV and REV co-infected host cells. Here, we reported that the Akt is more obviously activated and sustained in MDV and REV co-infected cells compare with in single virus infected cells. Furthermore, we revealed that RIOK3, but not PI3K, boosted Akt activity to promote the synergistic replication of MDV and REV, and we determined that the kinase activity of RIOK3 is required for the interaction between RIOK3 and Akt.

## Materials and methods

### Cells, viruses, antibodies, and inhibitors

Chicken embryonic fibroblasts (CEFs) were obtained from 10-day-old specific-pathogen free (SPF)-embryonated chicken eggs (Jinan Spafas Poultry Co., Ltd. Shandong, China). CEFs and chicken fibroblast cell line DF-1 were grown in Dulbecco’s modified Eagle medium (DMEM) supplemented with 10% foetal bovine serum at 37 °C in the presence of 5% CO_2_. The Md5 (very virulent, vv) strain of MDV (10^5^ plaque-forming units [pfu]/mL) and SNV strain of REV (10^4.2^ TCID_50_/mL) were maintained in our laboratory. The mouse anti-Flag, rabbit anti-HA (Sigma-Aldrich), and mouse anti-actin (Abcam) antibodies, rabbit polyclonal antibody (pAb) to Akt (BIOSS), rabbit polyclonal antibody (pAb) to *p*-Akt (BIOSS), rabbit polyclonal antibody (pAb) to 4EBP1 (ProteinTech), rabbit polyclonal antibody (pAb) to *p*-4EBP1 (BIOSS), and rabbit polyclonal antibody (pAb) to RIOK3 (Abcam) were used. The mouse monoclonal antibody anti-gp90 and rabbit polyclonal anti-pp38 were prepared in our laboratory [51].

Pretreatment with the PI3K inhibitor LY294002 and Akt inhibitor MK2206 (Beyotime Biotechnology) was conducted at optimum concentrations to avoid affecting CEF viability.

### Experiment design of REV and MDV co-infection

**Figure 1.**
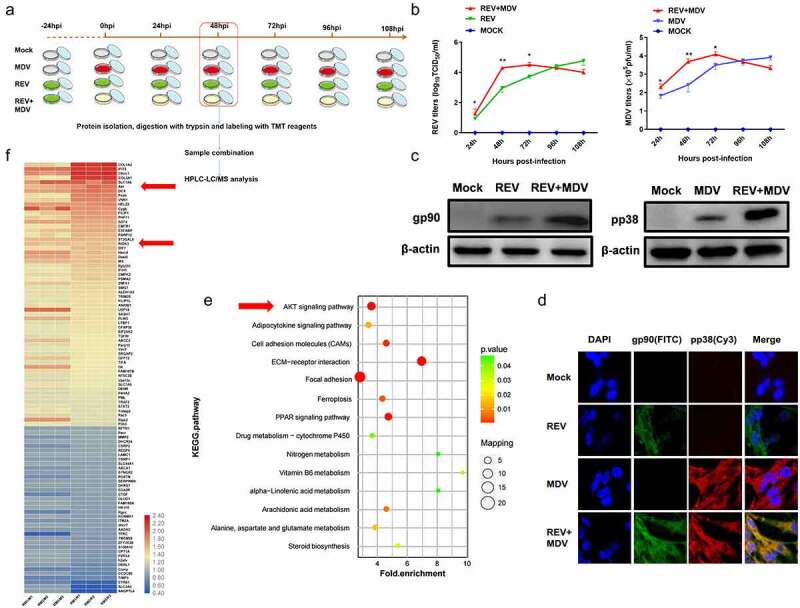
**MDV and REV enhanced mutual replication and activated the Akt pathway in co-infected cells**. (a) Co-infection model of REV and MDV and schematic of workflow for viral replication test, tandem mass tag peptide labelling coupled with LC-MS/MS analysis of CEF cells single-infected and co-infected with REV and MDV. (b) the kinetics of REV and MDV replication was tested in CEF cells. MDV-infected, REV-infected, and REV and MDV co-infected CEF cells or cell cultures were assessed for MDV pfu or REV TCID_50_ 24, 48, 72, and 96 hpi. The data represent the means of three independent experiments, with each experiment performed in triplicate. (c) Western blotting analysis of the MDV-pp38 and REV-gp90 expression. β-actin was used as an internal control to normalize the quantitative data. (d) Immuno-fluorescence analysis of REV and MDV infection in CEF cells. CEF cells were infected with MDV (MOI = 0.1, 100PFU) or REV (MOI = 1,1000 TCID_50_) for 48 h, and then MDV-pp38 (red) and REV-gp90 (green) were observed under an immunofluorescence microscope. (e) KEGG enrichment analysis of signalling pathways between REV and MDV co-infected CEF cells compared with single-infected CEF cells. Akt signalling pathway was indicated by the arrows. (f) Heatmap highlighting differences in the enrichment of cellular proteins between REV and MDV co-infected CEF cells compared with single-infected CEF cells, as indicated by the arrows. N, mock-infected CEF cells; R, REV-infected CEF cells; M, MDV-infected CEF cells; RM, REV and MDV co-infected CEF cells. Data are presented as the mean ± SD from three independent experiments. (One-way ANOVA, *p <0.05,**p <0.01).

We used three formats to establish the optimal co-infection model: initial infection with MDV then with REV 24 hours later; simultaneous MDV and REV infection; initial infection with REV then with MDV 24 hours later. The cell status was monitored, and the virus growth curve was evaluated using plaque-forming unit (PFU) and the 50% tissue culture infective dose (TCID_50_). The first two models caused cell death within 48 hours post-infection (hpi), and the two viruses did not show significant synergism. The third infection model showed significant synergistic replication of MDV and REV, and cell death occurred 72 hpi. Therefore, we selected the third co-infection model for subsequent experiments (Figure 1(a)). The optimal multiplicity of infection (MOI) was chosen to allow virus replication while causing minimal damage to CEF cells. Confocal imaging and western blotting were used to determine viral infection and proliferation. The mock, MDV- and REV-infected, and MDV+REV co-infected CEF cells were prepared for comparative TMT-LC-MS/MC analysis at the appropriate time intervals. Each sample comprised three technical replicates, and each experiment was conducted thrice.

### MDV and REV replication analyses

The replication of MDV and REV was measured using the PFU and TCID_50_ methods in the CEF cells at various time points, respectively. Briefly, 100 PFU of Md5 strain were inoculated into the CEF cells in 6-well plates and incubated at 37 °C with 5% CO_2._ The virus-infected CEFs were collected from 24 to 108 hpi to determine MDV replication in CEF cells, a series of two-fold dilutions was prepared into 96-well plates containing the CEFs in triplicate. Thereafter, count the number of plaques to determine viral titres from three independent experiments. 1000 TCID_50_ of REV SNV were inoculated into the CEF cells in 6-well plates. Infected cell cultures were harvested at 24, 48, 72, 96,108 hpi. The TCID_50_ per millilitre of REV was determined by an immunofluorescence assay, using the Reed-Muench formula. The infectious progeny were subsequently harvested in triplicate from REV-infected cell cultures.

The MDV and REV genome copy numbers was measured by real-time quantitative PCR (qPCR) as previously described [29,31].

### Confocal imaging

Cultured CEF cells and DF-1 cells were single infected or co-infected with MDV and REV in 15-mm culture dishes. DF-1 cells were transfected with the RIOK3-Flag, empty vector plasmid or the Akt-HA plasmid. For confocal imaging, firstly, cells were washed with cold PBS, and then cells were fixed with 4% paraformaldehyde for 30 minutes, permeabilized with 0.2% Triton X-100 for 15 minutes. Next, cells were blocked with 5% BSA for 1 h. Thereafter, the CEF cells were incubated with mouse anti-REV-gp90, FITC-labelled goat anti-mouse IgG or rabbit anti-MDV-pp38, Cy3-labelled goat anti-rabbit IgG diluted in PBS for 1 h. For DF-1 cells, mouse anti-Flag or rabbit anti-HA antibodies and FITC-labelled goat anti-rabbit IgG Cy3-labelled goat anti-mouse IgG secondary antibodies (BIOSS) were used. The overlapping of the two fluorescent marker colours appeared yellow. In addition, the nuclei of all infected cells were stained using DAPI (Beyotime Biotechnology). After washing five times with PBS, we examined the cells subsequently using an SP8 confocal laser scanning microscope (CLSM; Leica Microsystems, Wetzlar, Germany).

### Plasmid and shRNA construction

To construct the Akt expression plasmid, chicken Akt (GenBank accession no. NM_205055.1) was cloned into a pEX-3 (pGCMV/MCS/Neo) vector with the HA tag fused to its 3′ end to generate Akt-HA. Next, plasmids harbouring chicken RIOK3 (GenBank accession no. XM_004939781.4) were constructed by cloning the RIOK3 into pEX-3 with the Flag tag fused to the 3′ end. Thereafter, shRNAs specifically targeting chicken Akt (5′-GCA CAT TCA TTG GCT ACA AGG-3′) and RIOK3 (5′-GCA GAA GGA CCA TTT ATT ACA-3′) were designed and synthesized by GenePharma (Shanghai, China). RIOK3 kinase-dead mutant (K290A) was generated by site-directed mutagenesis using the QuickchangeTM kit (Stratagene). K290A primers were as follows:

5’-CGTGACAAATACATCGCCGATGACTACAGATTC-3’(F);

5’-GAATCTGTAGTCATCGGCGATGTATTTGTCACG-3’ (R).

Next, shRNA transfections in cells using RNAi-Mate (GenePharma) according to the manufacturer’s instructions. Finally, cells were harvested for further analysis 24 h post-transfection.

### Immunoprecipitation (IP) and western blot (WB) assay

IP was performed with cell lysates isolated from DF-1 cells. Briefly, DF-1 cells were cultured in 6-well plates one day and then transfected with the indicated plasmids. The cells were removed from the medium at 48 h post-transfection, washed with cold PBS, and then directly lysed on the plate with Lysis/Equilibration Buffer (TaKaRa). After centrifugation at 1000× *g* for 5 minutes, the supernatants were incubated overnight with the indicated antibodies at 4 °C. Thereafter, 20 μL protein G-sepharose beads (Roche Holding AG, Basel, Switzerland) were added to the sample. After incubated, the beads were washed five times with PBS, transferred to Eppendorf tubes with SDS loading buffer, and then boiled for 10 minutes before western blotting analysis.

For western blotting, cells were washed three times with PBS, lysed on ice with NP-40 lysis buffer (Beyotime Biotechnology). The samples were subsequently separated by SDS-PAGE and then transferred onto polyvinylidene difluoride membranes. Afterwards, the membranes were blocked using QuickBlockTM Blocking Buffer (Beyotime Biotechnology) for 15 minutes and incubated overnight with the indicated primary antibodies at 4 °C. The membranes were washed five times with Tris-buffered saline with Tween 20 and incubated secondary antibodies.

### HPLC fractionation and LC-MS/MS analysis

Cells infected with MDV and/or REV and the Akt-Flag IP samples were analysed using a high-performance liquid chromatography (HPLC) system (Thermo Fisher Scientific, Waltham, MA, USA) with an Agilent Zorbax 300Extend-C18 column. The tryptic peptides were dissolved on an EASY-nLC 1000 UPLC system (450 nL/min). Tandem mass spectrometry (MS/MS) was performed using a Q Exactive^TM^ HF-X system (Thermo Fisher Scientific). The MS/MS data were searched in the Uniprot-gallus FASTA database using the Maxquant search engine.

For protein abundance ratios, a 1.2-fold change was taken as the threshold, and a corrected *p*-value <0.05 was adopted to identify significant changes. To annotate protein pathways, Kyoto Encyclopaedia of Genes and Genomes (KEGG) database (http://www.genome.jp/kegg/tool/map_pathway2.html) was used.

### In vivo experiment

1-day-old SPF White Leghorn chickens were purchased from the poultry institute, Shandong academy of agricultural science. The 120 birds were randomly numbered and divided into four groups, then individually housed in negative pressure-filtered air isolators. On day one, The first group was inoculated with 2000 PFU of MDV in 200 µL diluent, while the second group was inoculated with 10^4^ TCID_50_ REV in 200 µL diluent. The third group was treated as follows: on day one, 2000 PFU of MDV in 200 µL diluent; on day four, 10^4^ TCID_50_ REV in 200 µL diluent. 30 chickens in control group were injected with DMEM. On 3, 7, 14, 21 and 35 days post-infection (dpi), four birds were randomly selected from each group and humanely euthanized. After necropsy, the spleen were collected for viral copies and Akt/p-Akt expression analysis. The DNA and RNA was extracted using the TIANGEN kit (TIANGEN, Beijing, China) and detected by qPCR. Animal experiments were conducted following protocols approved by the Committee on the Ethics of Animal Experiments of the Shandong Agricultural University.

### Statistical analysis

Statistical significance among groups was determined by one-way repeated measures ANOVA, and the data were presented as the means ± SD. Statistical significance was set at *p*-value <0.05.

## Results

### MDV and REV facilitate mutual replication and activate the Akt pathway in vitro

To determine the effects of MDV and REV co-infection on their replication *in vitro*, CEF cells were infected with REV (MOI = 1, 1000 TCID_50_) and then with MDV (MOI = 0.1,100 PFU) after 24 hours as described in the experimental design (Figure 1(a)). The viral titres of REV and MDV were quantified using TCID_50_ and the plaque assays from 24 to 108 hpi. FITC-labelled anti-gp90 and Cy3-labelled anti-pp38 antibodies were used to detect REV-gp90 and MDV-pp38 expression and localization by confocal laser scanning microscopy. The results indicated that the replication rate of REV or MDV was higher at 24 hpi (*p* < 0.05), 48 hpi (*p* < 0.01) and 72 hpi (*p* < 0.05) in the REV and MDV co-infected group compared with that in the MDV infected control group (24 hpi, 48 hpi, 72 hpi) or REV infected control group (48 hpi, 72 hpi, 96 hpi). The replication rate of MDV or REV was lower at 96 hpi and 108 hpi in REV and MDVco-infected cells (Figure 1(b)). The genome copy numbers of REV and MDV were measured by qPCR from 24 to 108 hpi. The results showed that the replication rate of REV or MDV was higher from 24 to 96 hpi and lower at 108 hpi in REV and MDV co-infected cells (Figure S1). Furthermore, viral protein expressions were evaluated by western blotting at 48 hpi, and the results showed that the protein expression levels of the gp90 of REV and pp38 of MDV in co-infected cells were significantly higher than those in single infected cells (Figure 1(c)). Meanwhile, the highly expressed gp90 and pp38 were co-localized in the cytoplasm of REV and MDV co-infected cells 48 hpi (Figure 1(d)). To determine which signalling pathway is regulated in the process of REV and MDV synergistic replication, the REV and MDV co-infected CEF cells 48 hpi, MDV-infected CEF cells 48 hpi and REV-infected CEF cells 72 hpi were selected for further TMT-LC-MS/MS analysis. KEGG and heatmap analysis showed that the Akt pathway was enriched (Figure 1(e)) and Akt was significantly upregulated (Figure 1(f), Supplementary Table S1, *p* ≤ 0.05, ratios ≥1.2 or ≤0.83) in REV and MDV co-infected cells. All the results suggested that REV and MDV synergistically increased viral replication and upregulated Akt pathway *in vitro*.

### Akt promotes synergistic replication of MDV and REV

**Figure 2.**
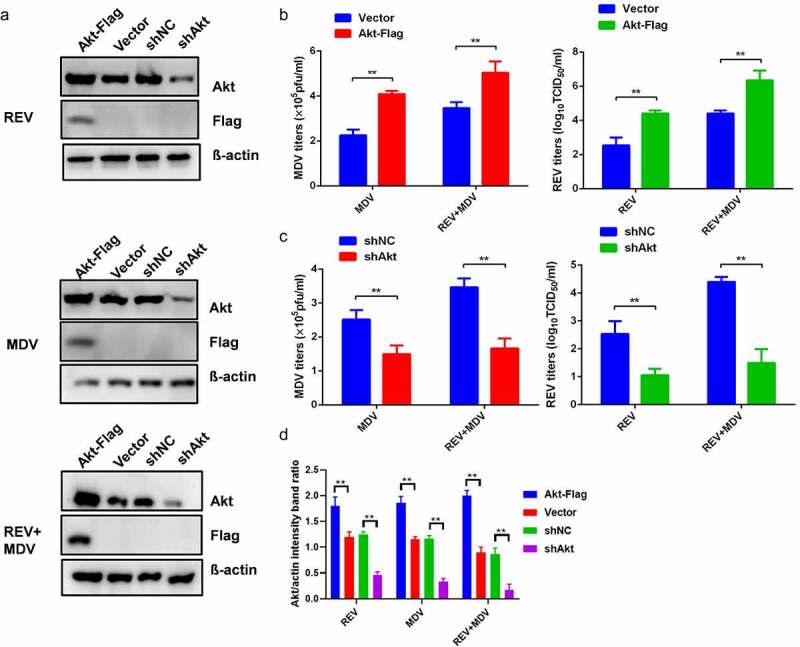
**Synergistic replication of REV and MDV is dependent on Akt**. (a) CEF cells were transfected with the empty vector or the Akt expression plasmid and shNC or shAkt. Twenty-four hours later, they were infected with MDV (MOI = 0.1,100PFU), REV (MOI = 1, 1000 TCID_50_), and REV and MDV. The REV and MDV viral titres were tested at 48 hpi (b, c). (d) Quantification of relative Akt band intensities to actin in (a). Data are presented as the mean ±SD from three independent experiments. Statistical analysis was performed using Student’s *t*-test (*, *p* <0.05; **, *p* <0.01).

To determine the effect of Akt on the synergistic replication of MDV and REV, Akt overexpression and interference were performed in CEF. CEF cells were transfected with Akt-Flag followed by infection with REV and/or MDV 24 h later, and the viral titres were determined by plaque and TCID_50_ assays 48 hpi. Akt overexpression remarkably enhanced the synergistic replication of MDV and REV (Figure 2(a,b,d)), while Akt knockdown significantly suppressed their synergism (Figure 2(a,c,d)).

### High Akt phosphorylation level is responsible for MDV and REV synergistic replication, independent of PI3K

**Figure 3.**
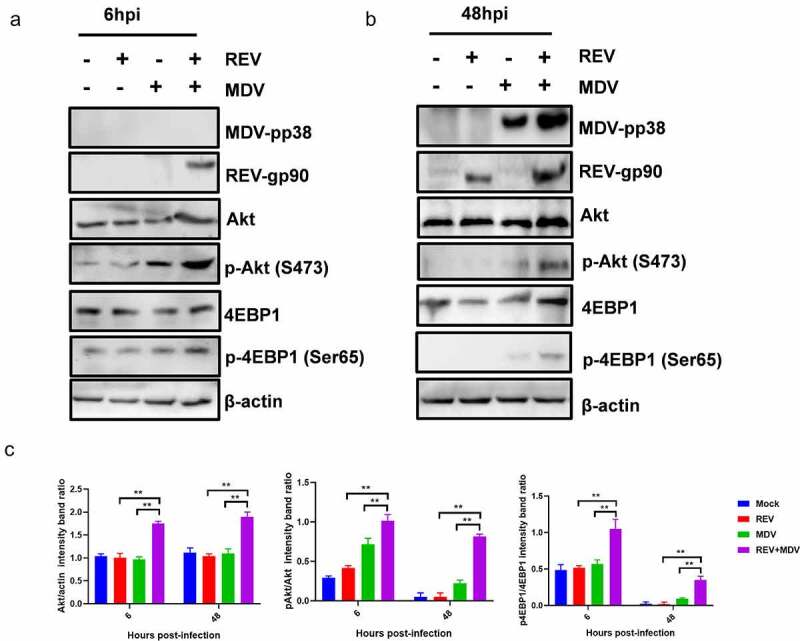
**Characterization of REV and MDV co-induced Akt pathway activation**. Different time points experiment in CEF cells co-infected or single-infected for 6 h (a) and 48 h (b) with REV and MDV in PBS or mock-infected. Cell lysates were prepared at the indicated time points and analysed by western blot analysis for Akt phosphorylation at Ser473, total Akt expression and 4EBP1 phosphorylation at Ser65, total 4EBP1 as well as MDV-pp38 and REV-gp90 expression; β-actin was used as a internal control. (c) Quantification of relative Akt band intensities to actin, relative pAkt band intensities to Akt and relative p4ebp1 band intensities to 4EBP1 in (a and b). Data are presented as the mean ±SD from three independent experiments. Statistical analysis was performed using Student’s *t*-test (*, *p* <0.05; **, *p* <0.01).

To determine how the Akt pathway affected the synergistic replication of MDV and REV, the expression and phosphorylation level of Akt and 4EBP1 were evaluated by western blotting. Cell lysates of the mock-, single- and co-infected CEF cells 6 and 48 hpi were prepared for western blotting. To determine viral infection, the pp38 (MDV) and gp90 (REV) protein expression levels were also evaluated by western blotting. The results revealed that phosphorylation status of Akt was increased in MDV/REV infected cells compared to the Akt phosphorylation levels in noninfected cells. Simultaneously, the phosphorylation level of Akt was increased in REV and MDV co-infected cells relative to that in single-infected cells 6 hpi (Figure 3(a,c)) and 48 hpi (Figure 3(b,c)). Concomitantly, the downstream Akt target 4EBP1 was more strongly phosphorylated in co-infected cells. The high levels of Akt phosphorylation subsequently persisted throughout both time points in REV and MDV co-infected cells while remaining undetectable in single-infected cells (Figure 3(a,b)). These data indicated that Akt activation was transient in MDV or REV single-infected cells but was sustained in REV and MDV co-infected cells. All the results suggested that MDV and REV synergistically upregulated Akt pathway *in vitro*.

**Figure 4.**
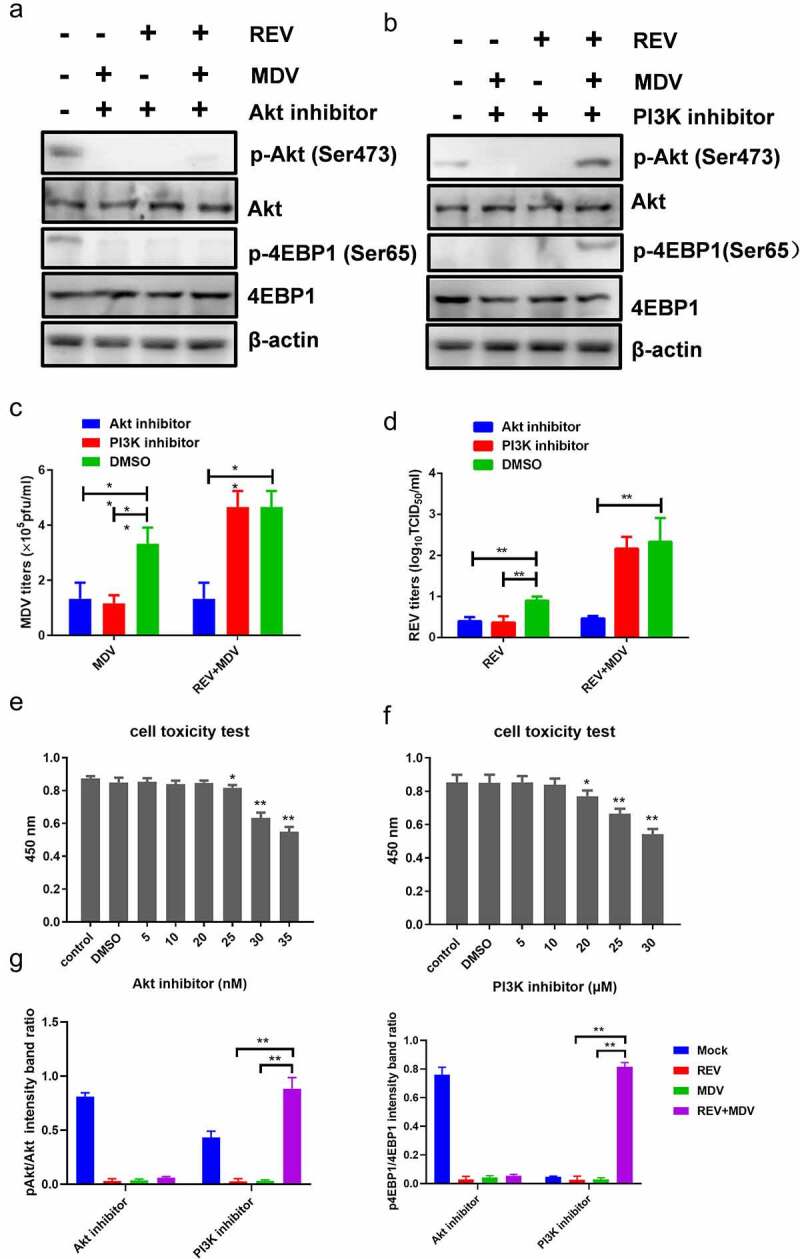
**REV and MDV co-infection activates Akt phosphorylation in a PI3K-independent manner**. (a and b) Akt inhibitor blocked MDV and/or REV-induced Akt phosphorylation. CEF cells were preincubated with MK2206 (25 nM) for 1 h, co-infected or single-infected with REV and MDV for 6 h, and cell lysates were analysed for the expression of pAkt (Ser 473), total Akt, and β-actin by western blotting. (c and d) Inhibition of PI3K does not influence viral replication of REV and MDV. CEF cells were co-infected or single-infected with REV and MDV (MOI = 1 for 1 h) and treated with 20 μM Ly294002 for another 6 h. (c) MDV plaque quantification. (d) TCID_50_ detection of REV titre. (e and f) Toxicity testing of Akt and PI3K-specific inhibitor. CEFs were treated with Akt inhibitor (5–35 nM), PI3K inhibitor (5–30 nM) and analysed for survival using the CCK-8 assay. (g) Quantification of relative pAkt band intensities to Akt and relative p4ebp1 band intensities to 4EBP1 in (a and b). The data represent the mean ± SD of three independent experiments. One-way ANOVA, (*, *p* <0.05; **, *p* <0.01).

To investigate whether Akt activation by MDV and REV was critical for the replication of the two viruses, CEF cells pretreated with or without Akt inhibitor MK2206 were infected with MDV and/or REV, and then the virus titres were determined 48 hpi. The CCK-8 assay showed that the optimum MK2206 concentration not affecting CEF viability was 25 nM (Figure 4(e)). Cell lysates were prepared to determine Akt and 4EBP1 phosphorylation 6 hpi using western blotting. Both Akt and 4EBP1 phosphorylation were decreased in MDV/REV infected and co-infected cells, indicating that the activation of Akt plays a critical role in the replication of the two viruses (Figure 4(a,g)). Furthermore, the replication of MDV or REV was significantly inhibited in both the single- and co-infected cells (Figure 4(c,d)). These results indicated that high Akt phosphorylation level promoted the synergistic replication of REV and MDV in co-infected cells.

To further understand the effect of PI3K, the major upstream molecule of Akt, on REV and MDV synergistic replication, we evaluated Akt and 4EBP1 phosphorylation after PI3K inhibition. The mock-, single-, and co-infected cells were maintained in a standard medium differentially treated with a specific PI3K inhibitor, LY294002. The CCK-8 assay showed that the optimum LY294002 concentration not affecting CEF viability was 20 µM (Figure 4(f)). Cell lysates were prepared 6 hpi and analysed by western blotting. On the one hand, in single-infected cells, Akt and 4EBP1 phosphorylation and the downstream readout of Akt activity were not detected upon LY294002 treatment (Figure 4(b,g)), confirming the efficacy of the inhibitor. On the other hand, the level of Akt and 4EBP1 phosphorylation in co-infected cells was reduced but not eliminated relative to that of single-infected cells. Furthermore, the replication of MDV or REV was not significantly inhibited in the co-infected cells, while virus replication was significantly reduced in REV and MDV single-infected cells 48 hpi (Figure 4(c,d,g)). These results indicated that PI3K inhibition did not influence viral growth in REV and MDV co-infected cells.

### RIOK3 is required for synergistic replication of MDV and REV

**Figure 5.**
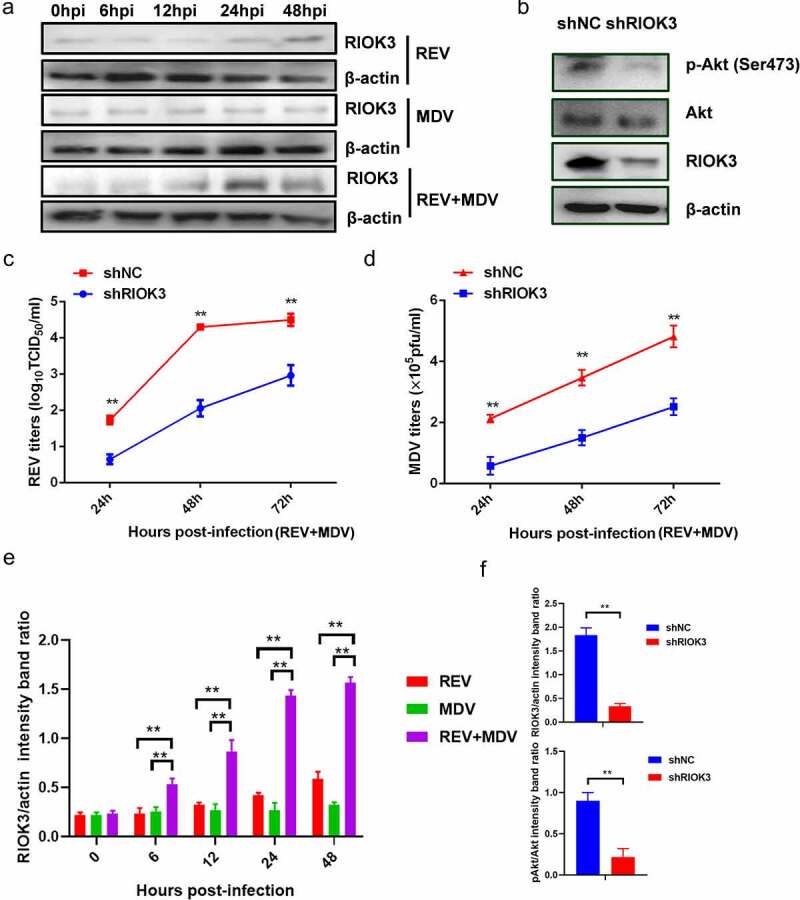
**RIOK3 is required for synergistic replication of REV and MDV**. (a) REV and MDV synergistically promote RIOK3 expression. The expression of RIOK3 during viral infection was assessed using western blotting. (b) RIOK3 knockdown decreased the level of Akt phosphorylation in REV and MDV co-infected cells. CEF cells were transfected with shRIOK3 and co-infected with REV and MDV for 6 h. Cell lysates were collected and analysed using western blotting with the indicated antibodies. (c and d) RIOK3 knockdown attenuates synergistic replication of REV and MDV. CEF cells were transfected with shRIOK3 and then co-infected with REV and MDV for 48 h. The MDV titre was measured using plaque quantification (c) and REV titre detected by TCID_50_ (d). (e and f) Quantification of relative RIOK3 band intensities to actin and relative pAkt band intensities to Akt (a and b). The data represent the mean ± SD of three independent experiments. One-way ANOVA, (*, *p* <0.05; **, *p* <0.01).

To further investigate the host factors responsible for Akt activation in REV and MDV co-infected cells, we performed IP-MS/MS to identify potential Akt-associated proteins. A construct containing the Akt gene and an empty vector were transfected into CEF cells which were then infected with MDV or/and REV. Thereafter, cells were subjected to lysis for the IP procedure using anti-Flag affinity gel 24 hpi, followed by 10% SDS-PAGE and MS analysis. The proteins present only in the IP product from REV and MDV co-infected cells but not in the IP product from MDV or REV infected cells were selected – we screened several proteins in the IP product treated with Akt. Of the screened proteins, a serine/threonine-protein kinase, RIO kinase 3 (RIOK3), attracted our attention because it was not only identified as an Akt-interacting protein in REV and MDV co-infected cells, but it was also significantly upregulated in REV and MDV co-infected cells (Figures 5(a,e) and 1(f)). To investigate whether RIOK3 was critical for the Akt phosphorylation and the replication of the two viruses in REV and MDV co-infected cells, CEF cells were transfected with eukaryotic expression plasmids expressing shRIOK3 and then infected with REV and MDV. The level of Akt phosphorylation was subsequently determined by western blotting 48 hpi. Virus titres were determined by the plaque and TCID_50_ assays 24, 48, 72, 96 and 108 hpi. The results showed that RIOK3 knockdown significantly decreased the level of Akt phosphorylation (Figure 5(b,f)) and suppressed the synergistic replication of REV and MDV in co-infected cells (Figure 5(c,d)). These data indicated that REV and MDV synergistically upregulated RIOK3 expression in vitro.

### RIOK3 increases Akt phosphorylation

**Figure 6.**
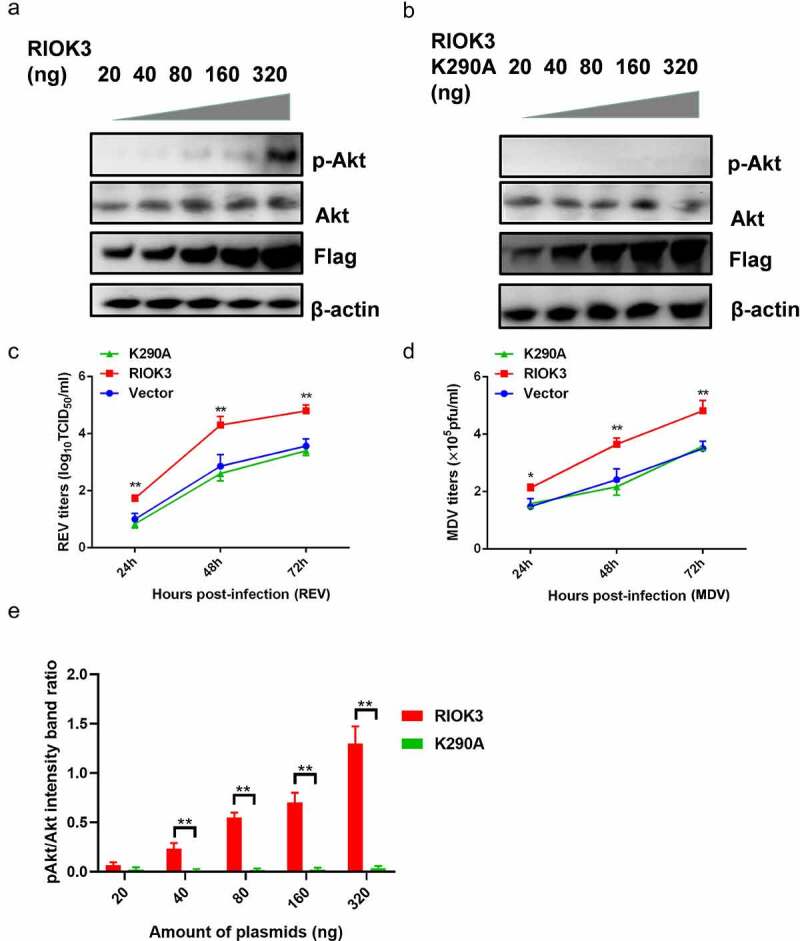
**Akt phosphorylation is dependent on the RIOK3 expression level**. (a and b) Increased expression level of RIOK3 increases Akt phosphorylation. DF-1 cells were transfected with the indicated amount of RIOK3 (a) or RIOK3 K290A (b). After 24 h, cell lysates were collected and analysed by western blotting with the indicated antibodies. (c) MDV plaque quantification. (d) TCID_50_ detection of REV titre. (e) Quantification of relative pAkt band intensities to Akt (a and b). All results are representative of three replicate experiments, and the data represent the mean ± SD. One-way ANOVA, (*, *P* <0.05; **, *P* <0.01).

Since RIOK3 is a kinase, it is possible to investigate the relevance of the association between the RIOK3 and Akt activity. To examine the role of RIOK3-mediated Akt phosphorylation in gene regulation, we transfected the constructed RIOK3 recombinant vector to assess Akt activity. Furthermore, to determine whether the kinase activity of RIOK3 is required for Akt phosphorylation, we constructed a RIOK3 kinase-dead mutant, K290A, in which the invariant lysine in subdomain II that is critical for ATP binding was mutated. The results showed that RIOK3 activated Akt activity in a dose-dependent manner (Figure 6(a,b,e)). As shown in Figures 6(c,d), RIOK3 overexpression enhanced the replication of REV and MDV. However, RIOK3-K290A did not affect virus replication in MDV- or REV-infected cells, suggesting that the kinase activity of RIOK3 is important for Akt phosphorylation and viral replication.

### RIOK3 interacts with Akt

**Figure 7.**
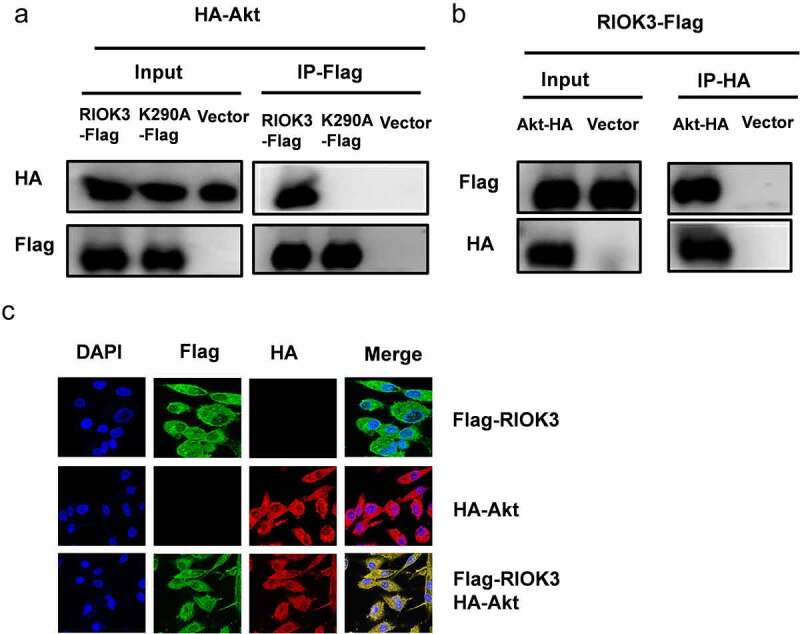
**RIOK3 interacts with Akt**. (a and b) Immunoprecipitation between RIOK3 and Akt. DF-1 cells were co-transfected with the RIOK3-Flag, RIOK3-K290A, and Akt-HA expression plasmids for 36 h, followed by a co-immunoprecipitation (co-IP) assay for RIOK3-Flag and Akt-HA using anti-HA (IP: HA) (a) or anti-Flag (Ip:flag) antibody (b). (c) Detection of co-localization of RIOK3 and Akt by IFA. DF-1 cells were transfected with the RIOK3-Flag or an empty vector, and 36 hpi, IFA was performed. The RIOK3 (green) and Akt (red) proteins were visualized with anti-Flag and anti-HA antibody. Cell nuclei (blue) were stained with DAPI. The areas of co-localization in merged images are shown in yellow.

To further investigate whether RIOK3 mediates the Akt-promoted synergistic replication of REV and MDV, we examined the physical association between RIOK3 and Akt. Cellular lysates from DF-1 cells co-transfected with RIOK3-Flag, RIOK3-K290A-Flag and Akt-HA were subjected to immunoprecipitation. Interestingly, our results demonstrated that Akt was efficiently co-precipitated with RIOK3-Flag, while failed associated with kinase-dead RIOK3-K290A, and reciprocally, RIOK3 could also be immunoprecipitated by Akt. These results suggested that the kinase activity of RIOK3 is important for its interaction with Akt (Figure 7(a,b)). In addition, to verify the interaction of RIOK3 with Akt, we examined the localization of RIOK3 and Akt in DF-1 cells by confocal microscopy, and the results revealed that Akt co-localized with RIOK3 in the cytoplasm (Figure 7(c)).

### RIOK-Akt pathway is activated in MDV and REV co-infected chicken

**Figure 8.**
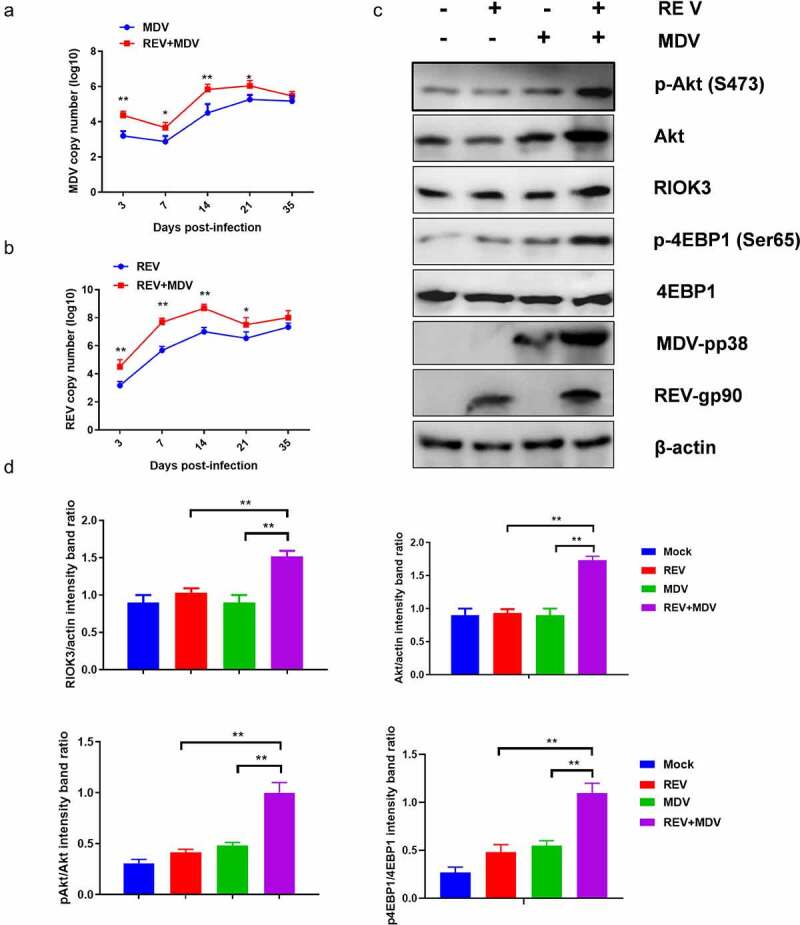
**Co-Infection chicken model of REV and MDV for viral synergistic replication test**. Replication kinetics of Md5 (a) or SNV (b) in REV and MDV coinfected chicken using qPCR. (c) Spleen of MDV/REV infected and co-infected chicken were collected at 14 dpiand analysed using western blotting with the indicated antibodies. (d) Quantification of relative RIOK3/Akt band intensities to actin, relative pAkt band intensities to Akt and relative p4ebp1 band intensities to 4EBP1 in (c. The data represent the mean ± SD of three independent experiments. One-way ANOVA, (*, *p* <0.05; **, *p* <0.01).

To determine whether MDV and REV facilitate mutual replication and activate the RIOK3-Akt pathway in vivo, we first determined the replication curves of MDV and REV in MDV/REV infected or co-infected chicken spleen. As shown in Figure 8(a,b), the replication rate of MDV or REV was higher from 3 dpi to 21 dpi in the REV and MDV co-infected chicken spleen compared with that in the MDV/REV infected control group and reached a peak at 14 dpi. Furthermore, western blotting analysis showed that the protein levels of RIOK3, Akt, *p*-Akt and *p*-4EBP1 in the spleen of REV and MDV co-infected chicken spleen were significantly higher than in those of REV/MDV infected chicken spleen at 14 dpi, respectively (Figure 8(c,d)). These data indicated that MDV and REV synergistically increased viral replication and activated RIOK3-Akt pathway in vivo.

## Discussion

Viruses usually activate intracellular PI3K/Akt signalling to promote viral infection and replication [36,37,39,40]. The activation of Akt can provide the benefits of increasing growth and suppressing apoptosiss [40]. Generally, the expression of Akt is strictly regulated in cells [52,53]. However, for many DNA or RNA viruses, viral infection benefit from increased Akt or phosphorylated Akt expression level. This phenomenon also occurs in more than one virus co-infected host. For example, It has reported that HIV Tat activates PI3K/Akt signalling and potentiates KSHV proteins oncogenic activity in KSHV and HIV co-infected hosts [54–56]. However, whether the synergistic replication between REV and MDV is regulated by PI3K/Akt pathway remains unclear.

In the present study, we observed that REV and MDV co-infection enhanced mutual replication *in vitro* and *in vivo*, indicating synergism between MDV and REV. To investigate whether the PI3K/Akt pathway was involved in this synergism, we conducted TMT-LC/MC analysis [51]. Heatmap and KEGG analysis showed that Akt protein levels were significantly upregulated, and Akt was involved in the main signalling pathway in REV and MDV co-infected cells. It has been proposed that the Meq protein of MDV could interact with PI3K, activating the PI3K/Akt pathway [50]. However, no reports have demonstrated the relationship between REV replication and the PI3K/Akt signalling pathway. Here, we demonstrated that high Akt expression was considerably associated with synergistic viral replication, and silencing Akt inhibited the synergistic replication of MDV and REV. Furthermore, we revealed that REV and MDV co-infection led to very strong and persistent Akt phosphorylation compared with the transient Akt phosphorylation observed in single-infected cells. We also revealed higher level of Akt, phosphorylation of Akt and 4EBP1 in REV and MDV coinfected chicken, indicating that Akt pathway activation likely supports synergistic viral replication of MDV and REV *in vitro* and *in vivo*.

It is well known that PI3K in virus-infected cells is sufficient to activate Akt [57,58]. For example, VP11/12 protein of herpes simplex virus 1 activates the PI3K/Akt transient phosphorylation [59] and influenza A virus NSP1 directly binds to the P85 subunit of PI3K activates Akt pathway [60]. However, CMV protein directly targets mTOR but not directly activate PI3K or Akt to support viral replication [61]. In the present study, PI3K inhibition had little influence on MDV/REV replication in co-infected cells, indicating that PI3K is not required for the synergistic replication of MDV and REV via Akt activation. Therefore, the host molecule/s mediating Akt activation in REV and MDV co-infected cells remain to be identified. To this effect, MS and Co-IP analysis revealed an interaction between RIOK3 and Akt.

RIOK3, a conserved atypical kinase, belongs to the RIO family, including three RIO kinases: RIOK1, RIOK2 and RIOK3 [62]. It has been shown that RIOK1 and RIOK2 play a crucial role in cell cycle progression [47,62], and RIOK3 is important for autophosphorylation [63]. In general, the phosphorylation and activation of Akt are thought to be mediated by PI3K activity. Therefore, we hypothesized that Akt is a substrate for RIOK3. As shown in the present study, the overexpression of RIOK3, but not of the kinase-dead mutant, induced Akt phosphorylation. Furthermore, induction of RIOK3-mediated Akt phosphorylation resulted in activation of 4EBP1 expression, indicating that RIOK3 alone can modulate cellular signalling pathways by activating Akt. We speculated that RIOK3 and Akt co-localize in co-infected cells since immunofluorescence and co-immunoprecipitation results demonstrated that RIOK3 and Akt were co-recruited.

Taken together, our results illustrate that MDV and REV activated a novel RIOK3-Akt signalling pathway to facilitate their synergistic replication. Further studies are needed to map the RIOK3 phosphorylation site of Akt to further explore the role of RIOK3-Akt pathway in viral replication, pathogenesis, and tumorigenesis during co-infection of MDV and REV.

## Supplementary Material

Supplemental Material

## Data Availability

The authors confirm that the data supporting the findings of this study are available within the article and its supplementary materials.
